# A Simplified Hollow-Core Photonic Crystal Fiber SERS Probe with a Fully Filled Photoreduction Silver Nanoprism

**DOI:** 10.3390/s18061726

**Published:** 2018-05-28

**Authors:** Youfu Geng, Yiwen Xu, Xiaoling Tan, Lina Wang, Xuejin Li, Yu Du, Xueming Hong

**Affiliations:** 1 Collage of Physics and Energy, Shenzhen Key Lab. of Sensor Technol., Shenzhen University, Shenzhen 518060, China; YiwenXu_szu@163.com (Y.X.); wanglina2017@email.szu.edu.cn (L.W.); lixuejin@szu.edu.cn (X.L.); duyu@szu.edu.cn (Y.D.); xmhong@szu.edu.cn (X.H.); 2 The Electronic Communication Department, Shenzhen Institute of Information Technology, Shenzhen 518172, China; tanxl@sziit.edu.cn(X.T.); 3 School of Science and Engineering, The Chinese University of Hong Kong (Shenzhen), Shenzhen 518172, China

**Keywords:** optical fiber sensor, refractive index measurement, micro-nano fiber, Mach-Zehnder interferometer, high sensitivity

## Abstract

In this paper, a simplified hollow-core photonic crystal fiber surface-enhanced Raman scattering (SERS) probe is presented. Silver nanoprisms are grown with a photoreduction method and account for the SERS, which have better electromagnetic enhancement than spherical silver nanoparticles at 785 nm. Due to the antiresonant reflecting guidance mechanism, the excited laser and SERS signal are effectively guided in such a fully filled hollow-core photonic crystal fiber SERS probe and complicated selective filling with target sample is avoided. Rhodamine 6G molecules are used as probe molecules and the simplified hollow-core photonic crystal fiber SERS probe is test. Detection of low concentration Rhodamine 6G down to 10^−8^ M is achieved with a short integration time of 300 ms.

## 1. Introduction

Surface-enhanced Raman scattering (SERS) fiber probes have attracted great attention in recent years for their remote online ability, compact structure and high sensitivity. Various structures emerge and present great potentials in surface science and engineering, single molecule analysis, biomedical application, food safety and environmental monitoring, etc. [1,2]. Optimized fiber probe structures could greatly improve the interaction area between excited laser and nanoparticles, e.g., by adopting fiber structures such as taper fiber [1,2,3], hollow core waveguide [4,5], D-shape fiber [6] and micro-structured fiber [7,8,9,10]. Among them, two types of micro-structured fiber have been used to construct fiber SERS probes, the total internal reflection photonic crystal fiber (TIR-PCF) and the hollow core photonic bandgap fiber (HC-PBF). In the HC-PBF SERS probes, the target liquid sample mixed with nanoparticles is selectively filled into the fiber core to form a large interaction area between sample and fiber core mode in an axial direction along the fiber. Therefore, it has much lower concentration detection limit than that of the TIR-PCF-based SERS probe, which uses the evanescent wave to excite the SERS signal. However, a pretreatment process, such as collapse of cladding air hole with arc discharge, is necessary to realize selective filling of sample into the fiber core, which makes the probe fabrication complicated.

The simplified hollow-core photonic crystal fiber (SHC-PCF) has a hexagonal hollow core and one layer of air holes [11]. The light is guided by the anti-resonant reflecting guidance mechanism (ARROW), that is, if the light is resonant and then guided in the high-index silica strut, the light can’t be guided in the fiber core, otherwise, if the anti-resonant condition of the silica strut is satisfied, the guided light could be confined in the low-index fiber core [11,12]. The SHC-PCF has been used to construct Fabry-Perot refractometers [13,14], long period gratings [15], fiber inclinometers [16] and pressure sensors [17,18].

SHC-PCF has a remarkable property, that is, the light could be guided even all the air holes are filled with liquid due to the fact the transmission windows only depend on the silica strut thickness and the surrounding refractive index. Therefore, a simple microfluidic sensor could be constructed without selectively filling the target liquid sample into the fiber core. In our previous work, an all-fiber reflecting fluorescent temperature probe was presented based on the SHC-PCF filled with CdSe/ZnS quantum dot aqueous solution, and a temperature sensitivity of around 126.23 pm/°C was obtained in a range of −10–120 °C [19]. In this paper, a SERS probe was fabricated based such a SHC-PCF with fully filled silver nanoprisms (AgPRs). It verified that the excited laser and SERS signal could still be guided effectively in such a fully filled SHC-PCF SERS probe, and hence, selectively filling of target sample with complicated fiber preprocessing was avoided in our experiment. The AgPRs grown with photoreduction method accounts for electromagnetic enhancement of SERS, and it shows good performance. Using Rhodamine 6G (R6G) molecules as probe molecules for such a SHC-PCF SERS probe, low R6G concentration down to 10^−8^ M is achieved with a short integration time of 300 ms.

## 2. Principle of the SERS Fiber Probe

**Figure 1:**
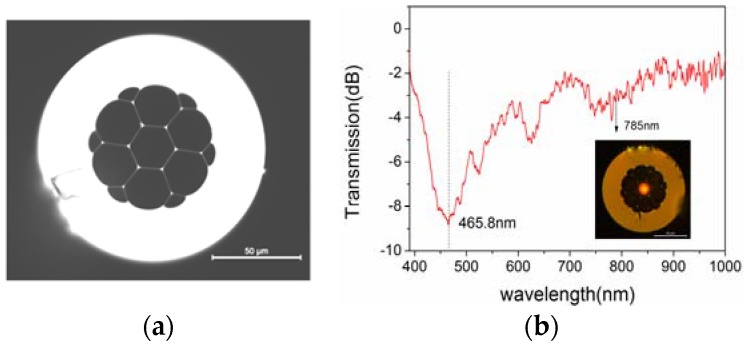
(**a**) Cross-section of the SHC-PCF; (**b**) Transmission spectrum of a 10-cm SHC-PCF from 400 nm to 1000 nm, the inset is a microscopy image of the SHC-PCF illuminated with a 785-nm laser.

The SHC-PCF used in our experiments is produced by the Yangtze Optical Fiber and Cable Corporation Ltd, Wuhan, China. A microscopy image of its cross-section is shown in Figure 1a. It consists of six large air holes and a central fiber core with a diameter of around 23.0 µm. The average thickness of silica struts around the central hole is measured to be around 0.235 µm. The diameter of the whole air-hole cladding is around 71.6 µm. According to the ARROW mechanism, such a fiber could guide the light coupled into the central hole. Figure 1b illustrates the transmitted spectrum of a 10-cm SHC-PCF from 400 nm to 1000 nm, the region of large transmission loss locates around 465.8 nm. The inset of Figure 1b shows the recorded output light spot as a 785-nm semiconductor laser is coupled into the central hole with a single-mode leading fiber.

**Figure 2:**
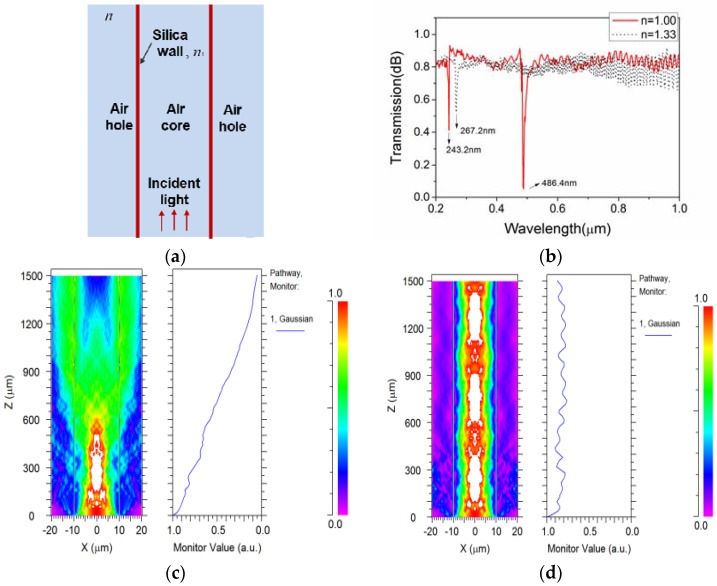
(**a**) Schematic of the SHC-PCF for simulation; (**b**) Transmission spectra from 0.2 µm to 1.0 µm as *n* = 1 and *n* = 1.33, respectively; (**c**) Side view of simulated 486.4-nm laser transmission in a 1.5-mm SHC-PCF without filling; (**d**) Side view of simulated 785-nm laser transmission in a 1.5-mm SHC-PCF with liquid filled.

The resonant wavelengths of SHC-PCF could be predicted with the expression as follows:

(1) $$\lambda_{m}=\frac{2t}{m}\sqrt{n_{1}^{2}-n^{2}}$$

where *λ_m_* is the *m*th-order resonant wavelength, *t* is the thickness of the silica strut, *n*_1_ is the refractive index of silica and *n* is the surrounding refractive index to silica struts. In order to get a clear view of transmission mechanism of SHC-PCF, a theoretical simulation is performed directly with the beam propagation method (BPM). The adopted two-dimensional schematic of SHC-PCF is as shown in Figure 2a. It consists of only two silica walls and a formed fiber core. The thickness of the silica struts is set to be 235 nm and the fiber core is around 23 µm, consistent with the real fiber structure.

Figure 2b presents the transmission spectra with and without liquid fully filled, that is, as *n* = 1.33 and *n* = 1, respectively. The resonant wavelength at 486.4 nm with air-hole PCF is close to the measured value which locates at 465.8 nm, and the deviation is mainly from the measurement errors of the fiber structure parameter. However, it shifts to 267.2 nm from 486.4 nm as SHC-PCF is filled with liquid. As shown in Figure 2c,d, the incident light spreads into the other holes and experiences large transmission loss at the resonant region, while it could be guided in the central core at the non-resonant region under the ARROW mechanism. It is suggested that both the excited laser and the SERS signal from 600 nm to 1000 nm could be effectively guided in a fully filled SHC-PCF.

## 3. Prepartion of AgPRs and Experimental Setup

The localized surface plasmon resonance (LSPR) wavelength of traditional silver nanospheres is from 350 nm to 400 nm, if a 785-nm excited laser is used, the electromagnetic enhancement at the surface the silver nanospheres is limited. Therefore, if the size and the shape of the silver nanoparticles change and make the excited laser wavelength lie in its plasmon resonance region, the SERS signal would be greatly enhanced. Hence, the photoreduction grown method is adopted and the AgPRs are prepared [20,21]. The synthesis of AgPRs is accomplished through two processes, the first is the precursor synthesis of spherical silver nanoseeds, the second is the nanoprism growing process with a 532-nm laser irradiation, and AgPRs grow gradually from the fusion of spherical silver nanoseeds under photoreduction catalysis.

**Figure 3:**
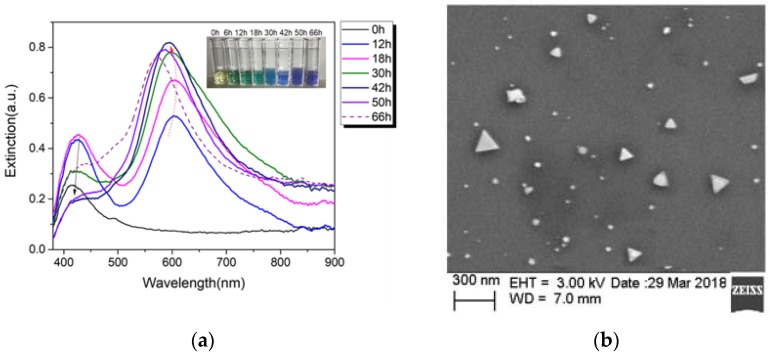
(**a**) The extinction of reaction solution during photoinduced conversion of silver nanospheres to nanoprisms, the inset shows the color evolution with irradiation times; (**b**) SEM image of the AgPRs synthesized by irradiation with a 532-nm laser, 30 h.

The detailed synthesis process is as follows: 10 mL of 10^−2^ mol/L silver nitrate solution was diluted with 90 mL deionized water, then 0.588 g sodium citrate as solid state powder was dissolved into the above diluted solution. With rapidly stirring, freshly prepared 10 mL of 10^−2^ mol/L NaBH_4_ was added into the above mixed solution dropwise. Thus, a yellow colored silver nanoseeds solution was successfully prepared and kept for 24 h. The size of the spherical silver seeds is around 3–5 nm. 5 mL of silver seed solution was selected to be illuminated by an all solid-state CW 532-nm laser with 50 mW power output. Under the laser irradiation, parts of the small spherical silver particles oxidatively dissolve into silver ion, and simultaneously, silver redox cycles are continuously conducted on the surfaces of those silver seeds with the anisotropic attachment of citrate, finally, the silver seeds gradually grow to the anisotropic structure of triangular silver nanoprism as the small spherical crystals are digested [19]. Once the spherical particles or nanoclusters are depleted, the reaction terminates. The UV-VIS transmission spectroscopy was used to monitor the growth process and the optical properties (LSPR) of silver nanoparticles in real time. After around 50 h, the absorption peak shifted to be around 625 nm in the UV-VIS extinction spectrum, and the color of the solution transformed into blue-green color as the inset of Figure 3a shows. With prolonged irradiation time, the absorption peak is blue-shift mainly due to the emergence of the truncated AgPRs and polygonal nanoplates. It presents a better SERS signal intensity of the AgPRs with 50 h irradiation than that of the AgPRs with other irradiation times. Therefore, those AgPRs are chosen to be mixed with target solution together to fill into the SHC-PCF further. Figure 3b shows the SEM image of the AgPRs synthesized by irradiation with a 532-nm laser to the 30 h. However, a small fraction of AgPRs with a dimension of 170 nm appeared, which could be avoided by precisely adjusting the pH value of the photoreduction solution or the concentration of reaction solution.

**Figure 4:**
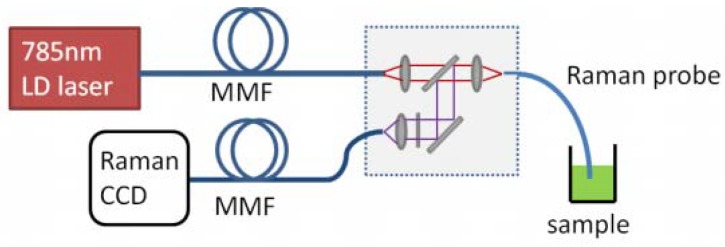
Experiment setup for SHC-PCF SERS probe test.

The experiment setup for SERS fiber probe test is shown as Figure 4. The excited laser is a 785-nm semiconductor laser with coupled output of a 105-μm core diameter multimode fiber (MMF), and the laser linewidth and maximum output power are around 0.1 nm and of 300 mW, respectively. It was coupled into a short piece of SHC-PCF with a 60× microscope objective. The focused light spot was around 30 µm, therefore, only the central hole was illuminated and used for guiding light. The SERS signal was collected by a 200-μm core diameter MMF and the Raman spectra were detected by an integrated charge-coupled device CCD Raman spectroscope (*i*-Raman plus 785H, B&W Tek Inc., Newark, DE, USA) with a Raman spectral range from 150 cm^−1^ to 2000 cm^−1^. The integration time was set to be 300 ms and the excited power is around 9.5 mW.

## 4. Experiment Results and Discussions

**Figure 5:**
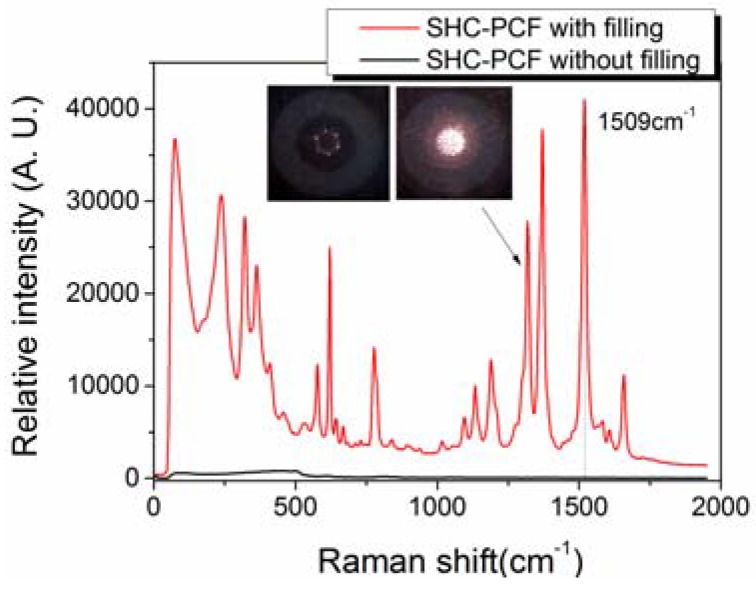
SERS signal of a 54-cm SHC-PCF with and without AgPRs filled. Integration time is 300 ms, and excited power is around 9.5 mW.

The R6G was chosen as target molecules to verify the SHC-PCF SERS probe. An aqueous solution is prepared by mixing with 170 µL 10^−3^ M AgPRs, 20 µL 10^−5^ M R6G and 10 µl 0.1 M NaCl, and the final concentration of R6G is 10^−6^ M. With the similar method, 10^−7^ M and 10^−8^ M R6G were also prepared, respectively. The NaCl accounts for enhancing attachment of R6G molecules to AgPRs. Firstly, the two ends of a short piece of SHC-PCF were cleaved, and the excited laser beam was delivered to the central hole from one end. Since the excited laser was guided in the air core, the Raman intensity of silica background was very low, which could greatly benefit the measurement of low concentration sample. Then the other end of the fiber was immersed into the mixed sample solution, and all the micro air holes would be fully filled by the well-known capillary force gradually, and finally, the sample solution came up to the laser delivering fiber end. Figure 5 shows the measured typical SERS spectra of a 54-cm SHC-PCF fully filled with target sample solution, the insets show the microscopy light spot at the fiber output end surface before and after filling with target liquid sample. Figure 5 presents a strong SERS characteristic peak at 1509 cm^−1^ for the 10^−6^ M R6G mixed sample solution with a short integration time of 300 ms.

**Figure 6:**
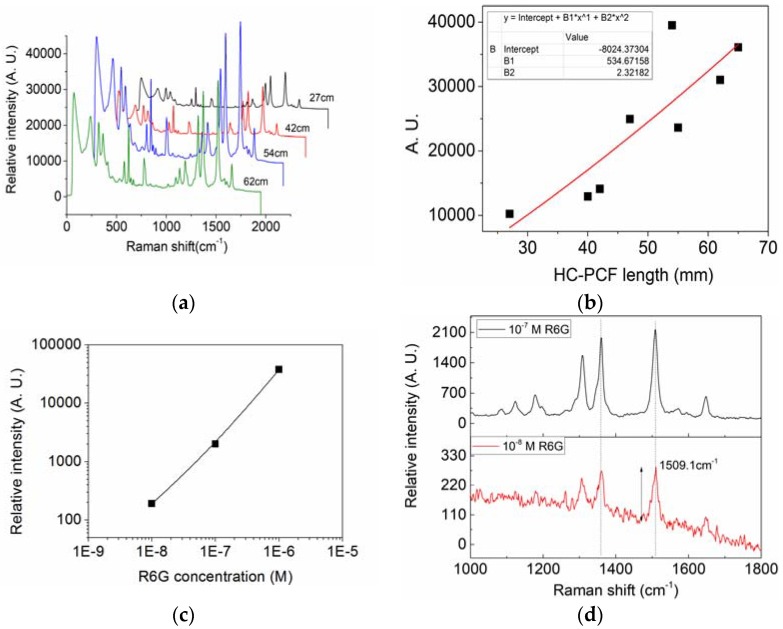
(**a**) Typical SERS spectra with filled SHC-PCF lengths of 27 cm, 42 cm, 54 cm and 62 cm, respectivley; (**b**) SERS signals versus lengths of SHC-PCF filled with AgPRs; (**c**) Relative SERS intensities versus R6G concentrations; (**d**) SERS spectra of a 54-mm SHC-PCF for 10^−7^ M and 10^−8^ M R6G, respectively.

Different SHC-PCFs with various fiber lengths are tested as filled with AgPRs fully. Figure 6a illustrates a waterfall map of the recorded SERS signals with filled SHC-PCF lengths of 27, 42, 54 and 62 cm, respectively. The CCD integration time and excitation power were still kept at 300 ms and 9.5 mW. It can be seen that clearly characteristic Raman peaks of R6G are present for all the filled SHC-PCFs, and the relative peak intensity at 1509 cm^−1^ increases with fiber length. Figure 6b shows the relative SERS intensities at 1509 cm^−1^ versus filled SHC-PCF fiber lengths, which illustrates a clear relationship of the increased SERS intensity with the SHC-PCF. The increased SERS signal with fiber length also suggests that, the SHC-PCF gives an important contribution of the Raman excitation and SERS signal enhancement with axially guided fiber modes. However, for the further elongated SHC-PCF, the SERS intensity decreases, which is due to the experienced transmission loss both for the excited laser and SERS signal in the aqueous solution filled SHC-PCF. It suggests that an optimized SHC-PCF length is needed for different target R6G concentrations. Relative SERS intensities and SERS spectra for different R6G concentrations were measured by 54-mm SHC-PCFs with a short integration time of 300 ms, as shown in Figure 6c,d. It can be seen that, the SERS intensity is linear with the concentration in log scale, and the characteristic Raman peak at 1509 cm^−1^ for low concentration R6G down to 10^−8^ M can still be distinguished from the noise background using this simple method and a short integration time, which can be comparable to the selective filling method. However, if the quality of the fiber is improved and the integration time is slightly elongated, the detection limit is expected to be further improved by this method.

## 5. Conclusions

In this paper, a SHC-PCF SERS probe was presented. Due to the ARROW mechanism, a fully filled SHC-PCF could effectively deliver the excited laser and SERS signal simultaneously, and thus complicated selectively filling of target aqueous solution sample into fiber air core was avoided. The AgPRs were grown with a photoreduction method and accounted for the SERS, which had better electromagnetic enhancement. R6G molcules were used as probe molecules and the SHC-PCF SERS probe was tested. The characteristic Raman peaks of R6G for low concentration R6G down to 10^−8^ M can still be distinguished from the noise backgroud with a short integration time of 300 ms. However, in the experiment, it is found that the fully filled SHC-PCF still has a relatively large transmission loss due to the inhomogeneous thickness and rough surface of silica struts. Therefore, if an improved SHC-PCF were used, much lower concentrations of target solution would be achievable.
